# Palladium(II)-Complexed *meso*-Tetra(4-pyridyl)porphyrin: Photodynamic Efficacy in 3D Pancreatic Cancer Models

**DOI:** 10.1021/acsomega.5c09619

**Published:** 2025-10-30

**Authors:** Edynara Cruz de Moraes, Lívia do Carmo Silva, Juliana Santana de Curcio, Alex Marchezini Graça, Alzir Azevedo Batista, Elisângela de Paula Silveira Lacerda, Pablo José Gonçalves

**Affiliations:** † Institute of Physics, 67824 Federal University of Goiás, Goiânia, Goiás 74690-900, Brazil; ‡ Laboratory of Oncogenetics and Medical Genetics, Institute of Biological Sciences, 67824 Federal University of Goiás, Goiânia, Goiás 74690-900, Brazil; § Department of Chemistry, 67828 Federal University of São Carlos (UFSCar), São Carlos, São Paulo 13565-905, Brazil; ∥ Center of Excellence in Hydrogen and Sustainable Energy Technologies (CEHTES), Goiânia, Goiás 74690-900, Brazil

## Abstract

Pancreatic ductal adenocarcinoma (PDAC) is among the deadliest malignancies, with limited therapeutic options and a highly unfavorable prognosis, largely attributed to the presence of a desmoplastic tumor microenvironment. In this study, the photodynamic efficacy of metalloporphyrin-based photosensitizers—palladium­(II)/diphosphine-coordinated *meso*-tetrapyridyl porphyrins, PS1 and PS2—were evaluated in both homogeneous and heterogeneous 3D models of PDAC composed of MIA PaCa-2 tumor cells and pancreatic cancer-associated fibroblasts. Photodynamic therapy was conducted using red light (λ = 635 nm) at doses ranging from 1.5 to 10 J/cm^2^, following a 90 min of incubation period. Among the tested compounds, PS2 demonstrated superior photodynamic performance in both models, exhibiting significantly lower IC_50_ values compared to PS1, thereby indicating enhanced phototoxicity. At a concentration of 3 μM, the IC_50_ value for PS2 was 1.3 ± 0.1 J/cm^2^, whereas for PS1 exhibited a markedly higher IC_50_ of 5.7 ± 0.06 J/cm^2^. This enhanced efficacy was attributed to sustained, dose-dependent generation of reactive oxygen species and increased cellular uptake. Notably, treatment with PS2 induced calreticulin exposure on the cell surface, indicative of immunogenic cell death. Organelle-specific analyses revealed mitochondrial hyperpolarization and decreased lysosomal fluorescence, suggesting selective subcellular accumulation and functional impairment.

## Graphical abstract

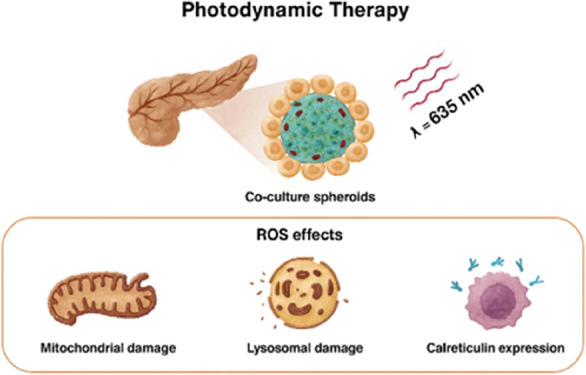

## 1 Introduction

Pancreatic ductal adenocarcinoma remains one of the most lethal cancers, characterized by limited therapeutic options and an extremely poor prognosis. Although recent advances have been made in diagnostic methods and therapeutic approaches, the five-year survival rate remains alarmingly low, around 9%.^1^ This unfavorable scenario is strongly associated with the presence of a highly desmoplastic tumor microenvironment, characterized by a dense extracellular matrix and the abundance of cancer-associated fibroblasts (CAFs), which can account for up to 90% of the tumor volume.^2^ This stromal structure acts as both a physical and biochemical barrier, hindering the penetration of therapeutic agents and limiting the effectiveness of approaches such as chemotherapy and immunotherapy. In this context, photodynamic therapy (PDT) has emerged as a promising alternative. This approach combines a photosensitizer (PS), light at a specific wavelength, and molecular oxygen to generate reactive oxygen species (ROS), promoting selective oxidative damage to tumor cells.^3^ In addition to its direct cytotoxic action, PDT has stood out for its ability to modulate the tumor microenvironment, reducing stromal stiffness and increasing tissue permeability. These effects contribute to improving the response to combination therapies, such as chemotherapeutics and immunotherapies.^4–6^

Another noteworthy aspect of PDT is its ability to activate the immune system through the induction of immunogenic cell death (ICD).^7^ ICD is characterized by the release of damage-associated molecular patterns (DAMPs), which act as cellular alarm signals, promoting the maturation of antigen-presenting cells (APCs) and the activation of CD8^+^ T lymphocytes.^8^ However, the success of this immune response directly depends on the amount of ROS generated. Thus, in hypoxic tumor microenvironments, ROS production is impaired, reducing the immunomodulatory efficacy of PDT and compromising its ability to promote antitumor immunity.^9^

Given these challenges, there has been growing interest in the search for more efficient photosensitizers capable of generating higher amounts of ROS and inducing ICD even under low oxygenation conditions. Therefore, the appropriate choice of PS is a determining factor for the success of PDT. Among the various photosensitizers studied, porphyrins and their derivatives stand out due to their photochemical and photophysical properties. These aromatic macrocycles exhibit strong absorption in the visible region, high efficiency in singlet oxygen generation, and preferential selectivity for tumor tissues. Moreover, their structure allows chemical modifications that improve solubility, selectivity, and photodynamic performance.^10^ The incorporation of metal ions into the porphyrin structure, especially those with catalytic properties or specific coordination geometry, has proven to be an effective strategy for enhancing the photodynamic properties of these molecules.^11^ Among these systems, porphyrins complexed with palladium (Pd) have attracted growing attention in PDT. The introduction of the Pd­(II) ion into the porphyrin core alters the electronic and photophysical properties of the compound, favoring intersystem crossing and the formation of the excited triplet state of the photosensitizer, which is essential for singlet oxygen generation.^12–14^ In addition to their photodynamic properties, palladium presents chemical similarities to platinum, a metal widely used in oncology, such as in cisplatin which suggests a potential cytotoxic effect independent of light activation.^15,16^ This dual mode of action makes palladium-based complexes promising candidates for photochemotherapy strategies. Another relevant aspect is the ability of palladium to catalyze the decomposition of hydrogen peroxide, an abundant metabolite in the tumor microenvironment into water and oxygen.^17^ This electrocatalytic effect contributes to the reversal of tumor hypoxia, a critical factor for enhancing the efficacy of photodynamic therapy (PDT).

A few years ago, our research group synthesized a series of metalloporphyrin-based photosensitizers—palladium­(II)/diphosphine-coordinated *meso*-tetrapyridyl porphyrins—which demonstrated intrinsic anticancer activity in the absence of light,^14^ as well as photodynamic inactivation properties against multidrug-resistant bacterial strains isolated from cases of bovine mastitis.^18^In the present study, we evaluated the photodynamic potential of two of these compounds, PS1 and PS2, in both homogeneous and heterogeneous three-dimensional (3D) models of pancreatic cancer. Taken together, these findings highlight the therapeutic versatility of these metalloporphyrins and support their potential as candidates for the development of innovative treatment strategies targeting pancreatic ductal adenocarcinoma, a malignancy that remains largely refractory to conventional therapies.

## 2 Materials and Methods

### 2.1 Samples

The compounds investigated in this study were synthesized according to previously described procedures in the literature.^14^ These compounds comprise a series of meso-tetra­(4-pyridyl)­porphyrins (TPyP) functionalized with four peripheral palladium­(II) complexes, each bearing a distinct 1,4-bis­(diphenylphosphino)­butane (dppb) (Figure 1A), and 1,1′-bis­(diphenylphosphino)­ferrocene (dppf) (Figure 1B). For clarity and brevity, these complexes are referred to as PS1 and PS2, respectively. The corresponding chemical structures are shown in the figure below.

**Figure 1:**
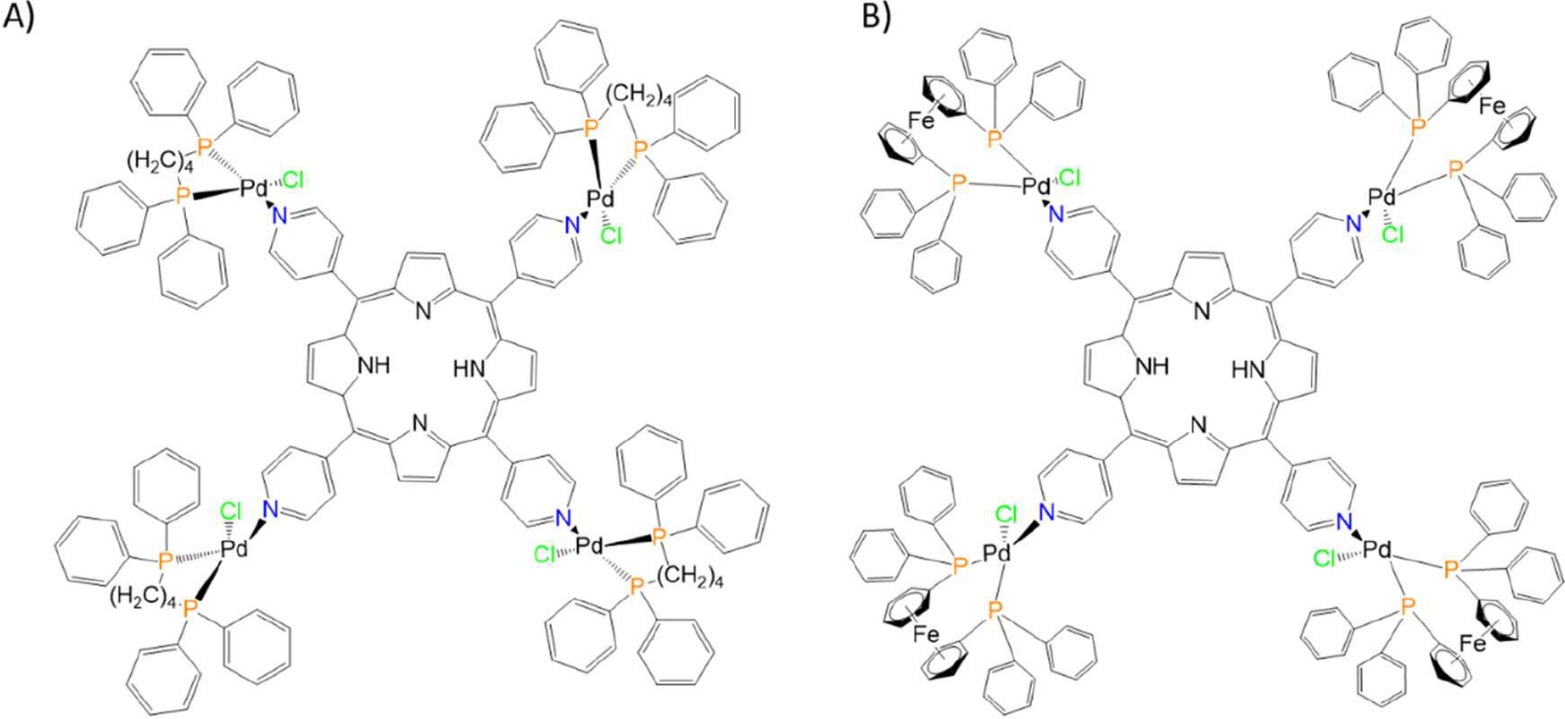
Chemical structures of the porphyrin-based complexes used in this study. (A) PS1 and (B) PS2.

### 2.2 Cell Lines

The human pancreatic ductal adenocarcinoma cell line Mia PaCa-2, expressing the fluorescent protein mCherry, and the pancreatic cancer-associated fibroblasts (PCAFs), expressing enhanced green fluorescent protein (EGFP), were kindly provided by the Wellman Center for Photomedicine, Massachusetts General Hospital (Dr. Tayyaba Hasan’s laboratory). All cell lines were cultured in Dulbecco’s Modified Eagle Medium (DMEM) supplemented with 10% fetal bovine serum (FBS) and 100 μg/mL penicillin-streptomycin, and maintained at 37 °C in a humidified incubator with 5% CO_2_.

### 2.3 Photodynamic Treatment in a Homogeneous 3D Tumor Model

The establishment of the 3D tumor model and subsequent analyses were carried out according to the protocol described by Saad.^2^ mCherry-MIA PaCa-2; 5 × 10^3^ cells/well were seeded into PrimeSurface 96U plates (96-well, round-bottom; Cat. No. MS-9096UZ, Sumitomo Bakelite Co., Ltd., Japan) to promote spheroid formation and incubate for 24 h prior to treatment. After this period, the spheroids were exposed to the photosensitizer complexes PS1 and PS2 for 90 min at final concentrations of 1, 2, or 3 μM. Upon completion of the incubation, the culture medium containing the photosensitizer was removed, and the spheroids were washed three times with fresh medium to eliminate excess compound before irradiation.

Irradiation was performed vertically, from the bottom of the microplates, using a diode laser source with a wavelength of 635 nm and an irradiance of 150 mW/cm^2^. The total light doses administered were 1.5, 3, 6, and 10 J/cm^2^. After irradiation, the plates were incubated for 72 h under controlled conditions (37 °C and 5% CO_2_) to allow the full development of PDT-induced cell death processes. Fluorescence imaging was subsequently performed to assess treatment outcomes in both tumor and stromal compartments

### 2.4 Photodynamic Treatment in a Heterogeneous 3D Tumor Model

To establish the heterogeneous 3D tumor model, mCherry-expressing MIA PaCa-2 cells and EGFP-expressing pancreatic cancer-associated fibroblasts (PCAFs) were coseeded into PrimeSurface 96U round-bottom plates (96-well format; Cat. No. MS-9096UZ, Sumitomo Bakelite Co., Ltd., Japan) at a total cell density of 5 × 10^3^ cells per well, using a 9:1 ratio of PCAFs to tumor cells (i.e., 90% PCAFs and 10% MIA PaCa-2). A 9:1 ratio of PCAFs to cancer cells was used to reproduce the fibroblast-rich composition typical of pancreatic tumors, where stromal cells can represent up to 90% of the tumor bulk, thus providing a physiologically relevant 3D microenvironment. The plates were incubated at 37 °C with 5% CO_2_ for 24 h to allow spheroid formation and stabilization prior to treatment.

Following spheroid formation, the culture medium was carefully removed and replaced with fresh medium containing the photosensitizer compound PS2 at final concentrations of 1, 2, or 3 μM. Spheroids were incubated with the photosensitizers for 90 min at 37 °C in the dark to allow compound uptake. After incubation, the medium was removed, and spheroids were washed three times with prewarmed DMEM to eliminate any excess noninternalized compound. Irradiation was then performed according to the parameters described in Section 2.3.

### 2.5 Reactive Oxygen Species (ROS) Generation

Mia PaCa-2 cells were seeded in black 96-well plates. On the following day, the cells were incubated with 3 μM of the photosensitizer PS1 or PS2 for 90 min. After incubation, the medium was removed, and the cells were washed with 1× PBS to eliminate excess noninternalized PS. Then, 5 μL of DCFDA probe solution from the DCFDA/H_2_DCFDA-Cellular ROS Assay Kit (ab113851, Abcam, Cambridge, UK) was added directly to each well containing PBS, to reach a final concentration of 10 μM. Subsequently, the cells were exposed to light doses of 1.5, 3, 6, and 10 J/cm^2^. The generation of ROS was assessed at 0, 1, and 2 h after PDT application by measuring fluorescence intensity using a microplate reader (Fluostar Omega, BMG Labtech, Ortenberg, Germany), configured with excitation and emission wavelengths of 485 and 535 nm, respectively. All experiments were performed in triplicate.

### 2.6 Uptake by Flow Cytometry

The uptake assay of the photosensitizers was performed using MIA PaCa-2 cells cultured as 3D spheroids in Corning Costar 24-Well Ultra-Low Attachment Plates (REF 3473, Corning Inc.). The spheroids were incubated with PS1 or PS2 (3 μM, 90 min, 37 °C, 5% CO_2_). After incubation, the medium was removed, and the spheroids were washed with 1× PBS. The spheroids were then dissociated using Cell Dissociation Buffer Enzyme-Free (Gibco, REF 13151-014) for 15 min at 37 °C, followed by centrifugation at 300 × *g* for 5 min. The resulting cell pellet was resuspended in FACS Buffer for analysis using a BD FACSAria II flow cytometer (BD Biosciences, San Jose, CA, USA). The fluorescence of the photosensitizers was detected in the APC channel (excitation: 650 nm, emission: 660 nm), and the data were processed using FlowJo software.

### 2.7 Calreticulin Detection

After treatment with 3 μM of the photosensitizer PS2 and irradiation at 1.5 and 10 J/cm^2^, MIA PaCa-2 cells were collected 72 h after photodynamic therapy, washed, and fixed with 4% paraformaldehyde (PFA) for 15 min at room temperature. For calreticulin detection, the protocol provided by the manufacturer of the anticalreticulin Alexa Fluor 488 antibody (Cell Signaling Technology, XP Rabbit, Cat. #12238S). For intracellular calreticulin detection, the cells were permeabilized with 0.1% Triton X-100 in PBS for 10 min on ice, followed by three washes with PBS to remove residual detergent. Next, the samples were incubated with the anticalreticulin Alexa Fluor 488 antibody (Cell Signaling Technology, XP Rabbit, Cat. #12238S) at the recommended dilution (1:50) for 30 min on ice, in the dark. After incubation, the cells were washed three times with PBS to remove unbound antibody. Finally, the stained cells were resuspended in 200 μL of FACS buffer (PBS with 2% FBS) and kept on ice until acquisition. Fluorescence data were collected using a BD FACSAria II flow cytometer (BD Biosciences, San Jose, CA, USA), detecting calreticulin fluorescence in the Alexa Fluor 488 channel (excitation: 499 nm; emission: 520 nm). The data were analyzed using FlowJo v10.8 software.

### 2.8 Organelle Damage Assessment

Organelle damage was evaluated 24 h post-treatment. For mitochondrial assessment, cells were incubated with MitoTracker Red (Thermo Fisher Scientific) at a final concentration of 100 nM in 1× PBS for 30 min at 37 °C in the dark. For lysosomal staining, cells were incubated with LysoTracker (Thermo Fisher Scientific) at a final concentration of 50 nM for 30 min at 37 °C in the dark. Cells were analyzed using a laser scanning confocal microscope (Olympus FV3000, Japan) with fluorescence detection at excitation/emission wavelengths of 561/580 nm for MitoTracker Red and 488/510 nm for LysoTracker. Images were acquired using ImageJ/Fiji software (National Institutes of Health, Bethesda, MD, USA). Organelle morphology was evaluated visually by qualitative examination of optical microscopy images, comparing treated and control groups for characteristic structural alterations associated with photodynamic damage.

### 2.9 Statistical Analysis

The software program GraphPad Prism was utilized to examine the statistical data collected from the experiment. The data was assessed using analysis of variance (ANOVA), and any variances in the mean were compared using the Tukey method. The findings were displayed as a mean with corresponding standard deviation. A significance level of *P* < 0.05 was used to determine the significance of any observed differences.

## 3 Results

### 3.1 Efficiency of Cellular Internalization

To compare the cellular internalization efficiency of the photosensitizer compounds, the uptake of PS2 and PS1 by MIA PaCa-2 cells was evaluated using fluorescence intensity measured by flow cytometry. As illustrated in Figure 2, the uptake of the PS2 compound (fluorescence intensity of 991.3) was approximately 57.6% higher than that observed for the PS1 compound (420), indicating a greater internalization efficiency of PS2 in these cells.

**Figure 2:**
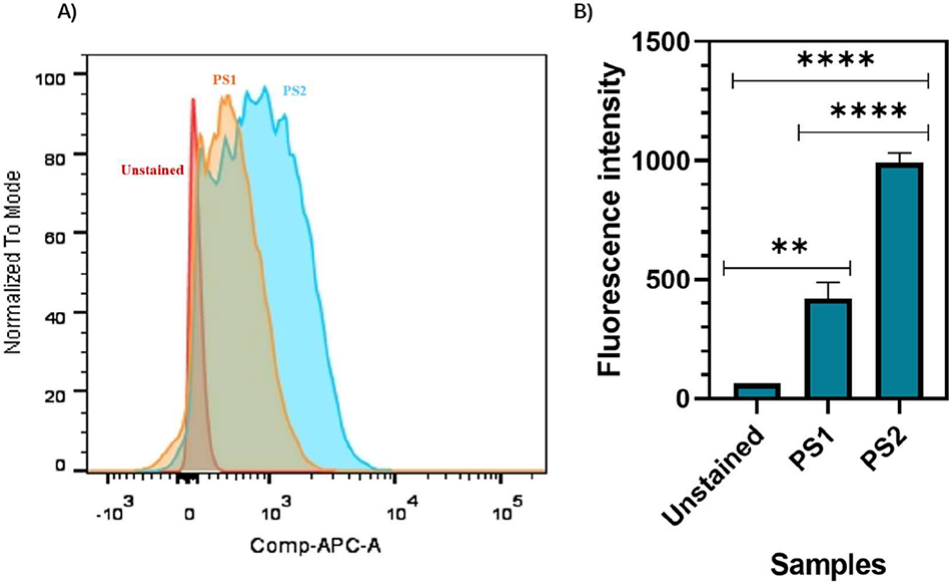
Cellular internalization of PS2 and PS1. Compounds in MIA PaCa-2 cells. (A) Flow cytometry histogram showing cellular uptake of the compounds PS2 and PS1 in MIA PaCa-2 cells after incubation within 3 μM of each compound for 90 min in the dark. (B) Intracellular uptake of the compounds PS1 and PS2 in MIA PaCa-2 cells after 90 min of incubation in the dark. Fluorescence intensity was measured to assess cellular internalization. Data are presented as mean ± standard deviation. **p < 0.01; **p < 0.0001.

### 3.2 Photodynamic Therapy Efficacy in a Homogeneous 3D Tumor Model

The efficacy of photodynamic therapy using PS1 and PS2 compounds was investigated in a homogeneous 3D tumor spheroid model of MIA PaCa-2 cells. Fluorescence imaging and dose–response analyses were performed 3 days after light exposure to assess phototoxic effects at varying compound concentrations. Fluorescence images and dose–response curves were acquired for concentrations of 1 μM (Figures S1 and S2), 2 μM (Figures S3 and S4), and 3 μM (Figures S5 and S6). Neither compound exhibited cytotoxicity in the absence of light, confirming a lack of dark toxicity. PS2 consistently showed greater phototoxic effects than PS1 at all tested concentrations. Specifically, at 3 μM, PS2 demonstrated an IC_50_ of 1.3 J/cm^2^, approximately 4.4 times lower than the 5.7 J/cm^2^ IC_50_ observed for PS1 (Table 1), highlighting its superior photodynamic efficacy in this 3D model.

**Table 1:** IC_50_ Values (J/cm^2^) for the Photosensitizers PS2 and PS1 at Concentrations of 1, 2, and 3 μM in 3D Tumor Spheroids Composed of mCherry-MIA PaCa-2 Cells

	**IC**_**50**_(J/cm^2^)		
**samples**	1 μM	2 μM	3 μM
PS1	9.8 ± 0.1	6.6 ± 0.6	5.7 ± 0.1
PS2	7.2 ± 0.8	3.7 ± 0.1	1.3 ± 0.1

### 3.3 PDT in a Heterogeneous 3D Tumor Model

The phototoxicity of PS2 heterogeneous spheroids were assessed by fluorescence imaging and dose–response curves, respectively, 3 days after the irradiation protocol. For the 1 μM concentration, the fluorescence image is shown in Figure S7, and the corresponding dose–response curve in Figure S8. For 2 μM, the fluorescence image is presented in Figure S9, and the dose–response curve in Figure S10. For 3 μM, the analyses are shown in Figure S11 (fluorescence image) and Figure S12 (dose–response curve). None of the compounds caused a decrease in spheroid viability in the absence of light, confirming the lack of dark cytotoxicity. The IC_50_ results presented in Table 2 demonstrate that both cell types responded similarly to the treatment, indicating that the photosensitizers induce photodynamic damage with comparable efficacy in pancreatic cancer cells and their associated fibroblasts. This effect is particularly evident at the 3 μM concentration, where the IC_50_ values were 2.3 J/cm^2^ for mCherry-MIA PaCa-2 cells and 2.6 J/cm^2^ for EGFP-PCAFs, respectively.

**Table 2:** IC_50_ Values (J/cm^2^) Obtained for the PS2 Tested at Different Concentrations (1, 2, and 3 μM) in 3D Tumor Spheroids^a^

	**IC**_**50**_(J/cm^2^)		
**cell line**	1 μM	2 μM	3 μM
mCherry-MIA PaCa-2	>10	4.7 ± 0.4	2.3 ± 0.1
EGFP-PCAFs	>10	6.1 ± 0.5	2.6 ± 0.5

a Viability was assessed in two cell lines: mCherry-MIA PaCa-2 (human pancreatic ductal adenocarcinoma) and EGFP-PCAFs (pancreatic cancer-associated fibroblasts).

### 3.4 Kinetic Analysis of ROS Generation Induced by Pd Complexes

To investigate the photosensitizing efficiency and the temporal dynamics of reactive oxygen species generation, we evaluated the dose-and time-dependent response of PS2 and PS2 complexes upon light irradiation. This analysis aimed to determine the stability and persistence of ROS production over time, as well as to compare the photodynamic performance of both compounds under varying light doses.

The PS2 compound exhibited a distinct response profile. At the initial time point (0 h), only the higher light doses (6 and 10 J/cm^2^) induced a significant increase in ROS production, with increments of 47 and 68%, respectively, compared to the control (Figure 3A). These levels remained constant up to 1 h, with the 10 J/cm^2^ dose reaching a peak of 154% relative to the control. However, at 2 h, a marked decrease in fluorescence was observed across all doses, indicating a reduction in ROS generation capacity. The lower doses (1.5, 3, and 6 J/cm^2^) showed values like the control, while the 10 J/cm^2^ dose, although still effective (62%), showed a significant reduction compared to its peak value (154%). In contrast, the PS2 compound displayed a dose-dependent and sustained response, with mean fluorescence increases of approximately 38, 77, 112, and 120% at 1.5, 3, 6, and 10 J/cm^2^, respectively (Figure 3B). These values remained stable over the course of 2 h, indicating continuous ROS generation.

**Figure 3:**
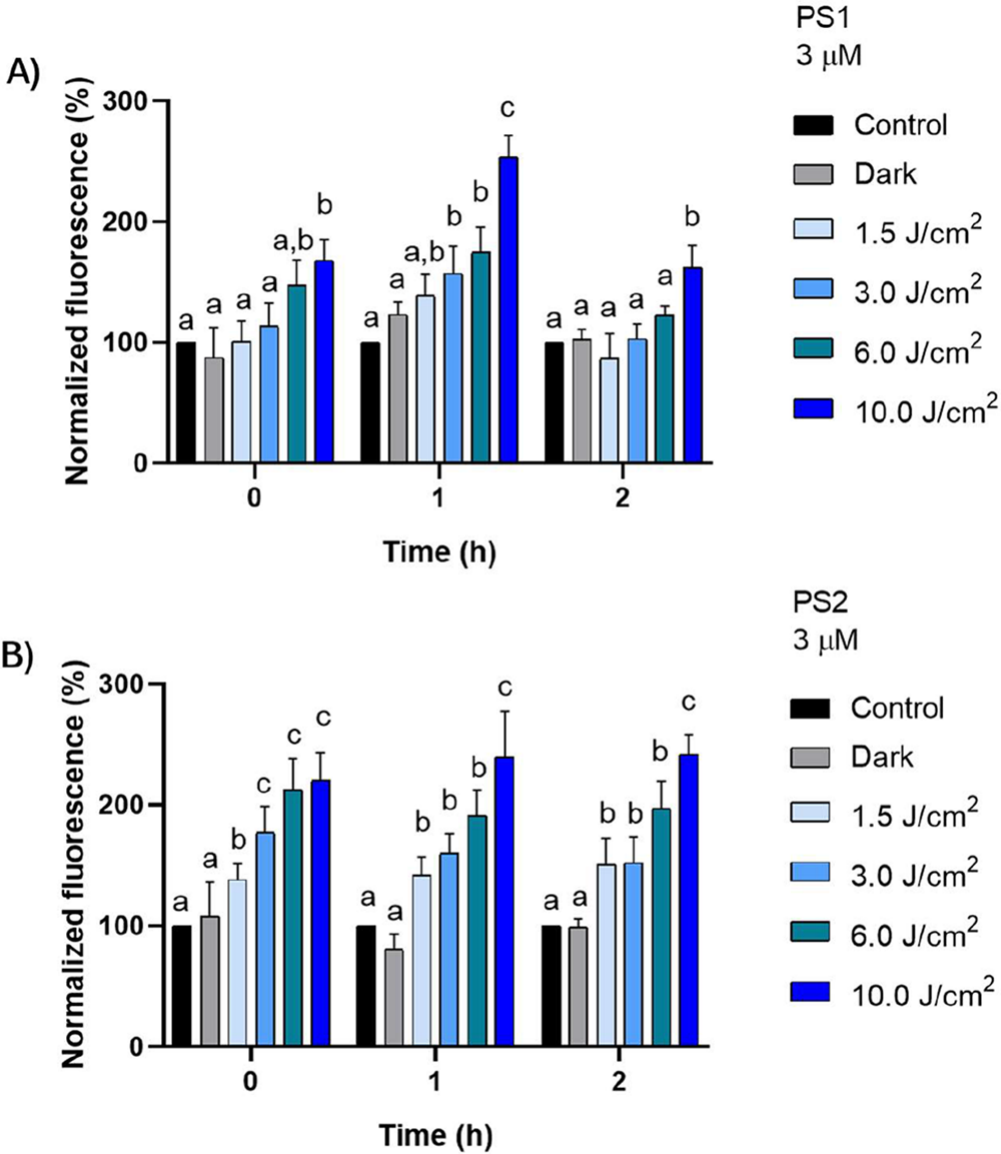
ROS generation induced by the compound (a) PS1 (b) PS2 (3 μM) in cells treated under different light conditions. Cells were exposed to light doses of 1.5, 3, 6, and 10 J/cm^2^, as well as to dark and untreated (control) conditions. Different letters indicate statistically significant differences between groups (*p* < 0.05).

### 3.5 Lysosomal and Mitochondrial Effects of Complexes

Fluorescence microscopy was employed to evaluate the effects of the PS2 and PS1 complexes on the integrity and functionality of specific organelles. Mitochondrial staining with MitoTracker (red), depicted in Figure 4, showed increased fluorescence intensity in treated cells, particularly with the PS2 complex, indicating possible mitochondrial hyperpolarization or differential dye retention consistent with treatment-induced functional alterations.

**Figure 4:**
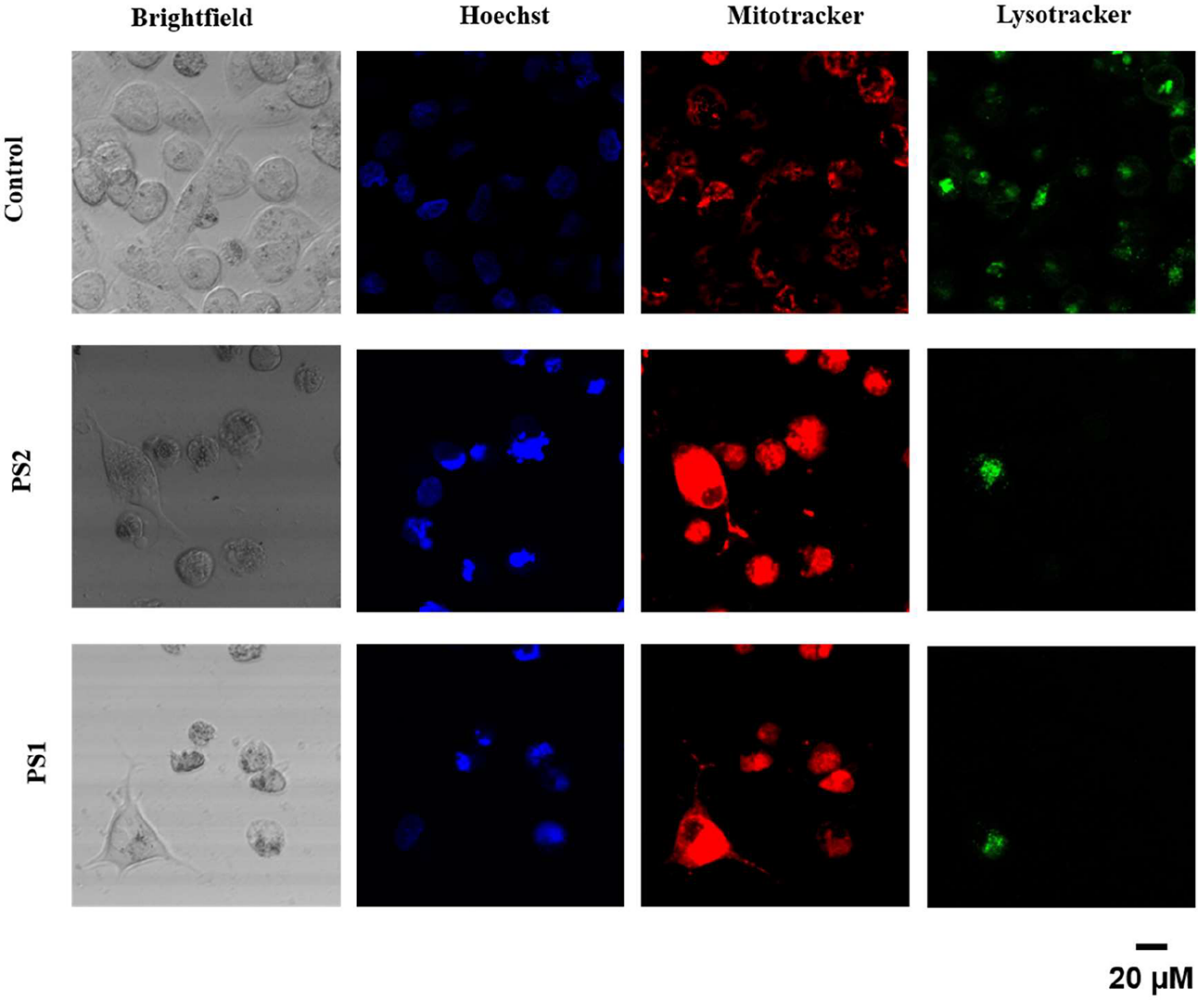
Fluorescence microscopy analysis of mitochondrial (red), lysosomal (green), and nuclear (blue) integrity after complex treatment.

In contrast, lysosomal staining with LysoTracker (green) exhibited a significant reduction in fluorescence in cells treated with both complexes compared to the control (Figure 4), suggesting compromised lysosomal membrane integrity or decreased compartmental acidity

### 3.6 Immunogenicity Induced by Photodynamic Therapy with PS2

The effect of different light doses on the exposure of calreticulin in MIA PaCa-2 cells treated with PS2, as an indicator of immunogenic cell death, was investigated. A significant increase in calreticulin exposure was observed in response to the light dose, as evidenced by the representative fluorescence histogram (Figure 5A) and the quantitative analysis (Figure 5B). The 10 J/cm^2^ dose resulted in a fluorescence intensity of 5117.3, representing an increase of approximately 84% compared to the nonirradiated control (0 J/cm^2^; 833). In contrast, the 1.5 J/cm^2^ dose (802) showed no statistically significant difference compared to the control, indicating a dose-dependent effect.

**Figure 5:**
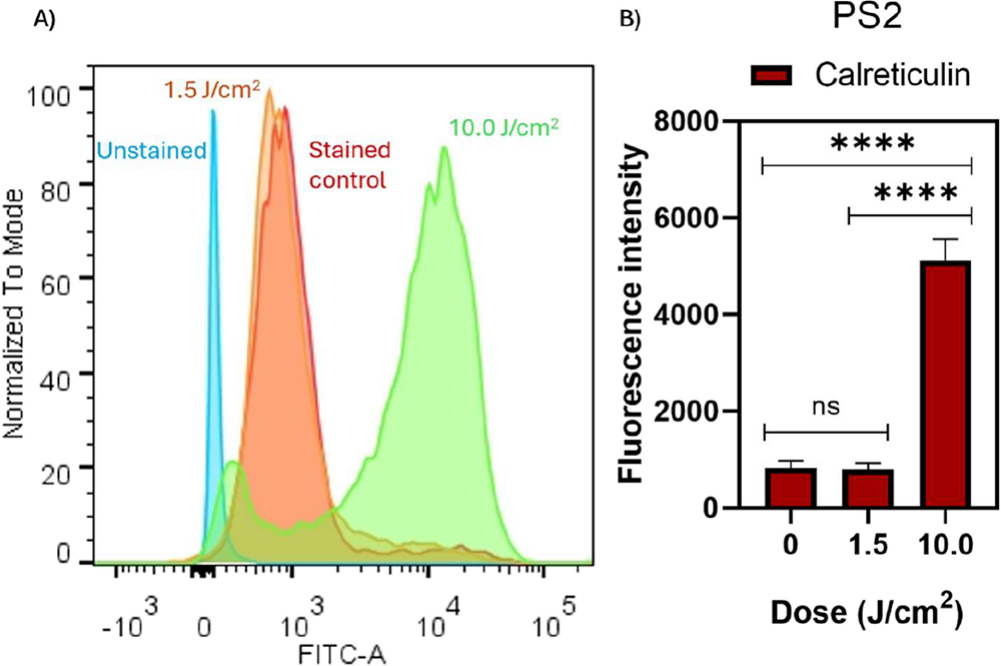
Calreticulin exposure induced by the PS2 under variable light irradiation. (A) Representative fluorescence histogram obtained by flow cytometry showing calreticulin exposure on the cell surface of MIA PaCa-2 cells treated with PS2 (3 μM) under different irradiation conditions. (B) Quantification of calreticulin exposure after treatment with PS2 (3 μM) and irradiation with increasing light doses (0, 1.5, and 10 J/cm^2^). ns: not significant; ****p < 0.0001.

## 4 Discussion

Given this potential, the present study investigated the photodynamic activity of two novel photosensitizers—PS2 and PS1—using homogeneous and heterogeneous three-dimensional PDAC models. Among the compounds evaluated, PS2 demonstrated superior photodynamic efficacy under all tested conditions. This enhanced activity can be attributed to a combination of structural and physicochemical features. One of the main contributing factors is the presence of a ferrocene group, which introduces iron ions capable of participating in intracellular redox reactions. These iron ions catalyze the decomposition of hydrogen peroxide (H_2_O_2_) through the Fenton reaction, leading to the formation of hydroxyl radicals (^·^OH)—highly reactive and cytotoxic species that contribute significantly to oxidative stress and cell death.^19^

In homogeneous spheroids composed solely of tumor cells, treatment with PS2 at 1 μM and 10 J/cm^2^ reduced cell viability to approximately 20%. This outcome is remarkably like that reported by Saad et al.,^2^ who employed photoimmunotherapy with cetuximab–BPD conjugates and achieved comparable cytotoxicity levels. Notably, BPD (verteporfin) is a clinically approved second-generation photosensitizer characterized by absorption in the red region (∼690 nm), high singlet oxygen quantum yield, and rapid clearance. When stromal components were introduced into the 3D system, thereby generating heterogeneous spheroids with 90% pancreatic cancer-associated fibroblasts (PCAFs), the efficacy of PS2 became even more evident. At 2 μM and 10 J/cm^2^, the compound induced complete spheroid eradication. In comparison, De Silva et al.^4^ reported ∼10–15% residual viability using a more complex triple-receptor targeted photoimmunonano conjugate loaded with BPD, at 1 μM and 100 J/cm^2^, in a model containing 50% PCAFs. Despite the differences in light dose, drug formulation, and stromal proportion, our results highlight that a nontargeted photosensitizer can reach comparable or even superior efficacy under highly desmoplastic conditions.

Another crucial aspect observed was the significantly higher intracellular uptake of PS2, possibly resulting from its greater lipophilicity, which facilitates passive diffusion across the lipid bilayer of the plasma membrane. This interpretation is supported by previous reports demonstrating that increased lipophilicity is a key determinant of photosensitizer uptake and intracellular distribution.^14^ In addition, the presence of a ferrocenyl moiety in the diphosphine ligand is likely to enhance the interaction with cellular membranes and promote uptake. Recent studies on ferrocene-based nanomedicines corroborate this hypothesis, showing that ferrocenyl groups can improve cellular internalization and bioavailability of therapeutic agents.^20^ Together, these findings suggest that PS2 possesses intrinsic structural features that favor cell entry, reducing the need for additional targeting strategies such as antibody or nanoparticle conjugation.

The kinetic analysis of ROS generation revealed distinct photoreactivity profiles for PS2 and PS1 complexes upon light exposure PS2 exhibited a dose-dependent and sustained ROS production, with fluorescence intensities increasing progressively with the light dose and remaining stable over a 2 h period. This pattern suggests a consistent and prolonged activation of the photosensitizer and aligns with previous reports indicating that ferrocenyl-containing palladium complexes, such as PS2, possess enhanced stability and electron-transfer capabilities that favor prolonged ROS generation.^21^ The ferrocenyl moiety in PS2 is known to stabilize radical intermediates and facilitate redox cycling, enhancing the efficiency of singlet oxygen or superoxide generation.^22^ On the other hand, the diphenylphosphinobutane (dppb) ligand, although potentially contributing to initial ROS formation, may not support sustained ROS release under extended light exposure, potentially due to ligand degradation or reduced triplet state lifetime.

The generation and persistence of ROS are crucial factors for the efficacy of PDT. ROS are responsible for oxidative damage to essential cellular components, including lipids, proteins, and DNA,^23^ leading to cell death through mechanisms such as apoptosis, necrosis, or immunogenic cell death.^24^ In the case of the PS2 complex, which exhibits sustained and dose-dependent ROS generation, this characteristic is expected to promote prolonged oxidative stress within the tumor microenvironment. This continuous stress can lead to increased induction of apoptosis and signs of immunogenic cell death, such as calreticulin exposure and the release of damage-associated molecular patterns (DAMPs), which activate antitumor immune responses.^25,26^

The sustained and dose-dependent ROS production observed for PS2 represents a significant advantage over several clinically relevant photosensitizers, such as chlorins, phthalocyanines, and BPD (verteporfin), which typically exhibit an initial burst of ROS generation followed by a rapid decline. This transient behavior can limit the extent of oxidative damage, particularly in tumor models characterized by dense stroma and hypoxic niches. In contrast, palladium–porphyrin complexes have been reported to produce ROS more efficiently and with greater stability, as demonstrated by Tisoco et al.^12^ and Deng et al.^13^ Our findings agree with these reports, highlighting that the ferrocenyl-containing palladium complex maintains continuous ROS release over time. This persistent oxidative stress is expected to amplify cell death pathways and favor immunogenic mechanisms, providing a therapeutic advantage in the treatment of pancreatic ductal adenocarcinoma.

Our findings using MitoTracker Red and LysoTracker markers further confirm that PDT induces coordinated damage to vital cellular organelles, undermining cellular homeostasis and activating multiple programmed cell death pathways. The marked increase in mitochondrial fluorescence after treatment indicates a significant loss of mitochondrial membrane potential (ΔΨ_m_), reflecting severe mitochondrial dysfunction characteristic of the intrinsic apoptotic pathway. This dysfunction is likely mediated by ROS acting directly on the mitochondrial membrane, causing cytochrome c release and triggering the caspase cascade leading to apoptosis. Simultaneously, reduced lysosomal fluorescence suggests lysosomal membrane permeabilization (LMP), which can release hydrolases into the cytoplasm. LMP contributes not only to necrotic cell death but also potentiates immunogenic cell death, amplifying the biological impact of PDT. Importantly, LMP can also induce inflammatory responses, a mechanism particularly relevant in therapies aimed at stimulating antitumor immunity. This lysosome-derived inflammatory signaling often works in concert with the exposure of immunogenic markers on the cell surface, such as calreticulin, which further promote immune system activation.^27–29^

Calreticulin acts as DAMPs, functioning as an “eat-me” signal for antigen-presenting cells, thereby promoting the recruitment and activation of dendritic cells, followed by the activation of T lymphocytes.^30^ According to the literature, the release of DAMPs such as calreticulin may induce a relevant immunomodulatory effect by promoting the conversion of an immunologically “cold” tumor microenvironment—characterized by low immunogenicity—into a “hot” microenvironment, more permissive to T cell infiltration and activation.^31^ This phenomenon is particularly important in tumors like PDAC, which are known for their immune evasion.

Additionally, studies have shown that iron may influence macrophage polarization, favoring the pro-inflammatory M1 phenotype, which is associated with more immunogenic tumor environments.^32^ Although calreticulin exposure is a classical marker of immunogenicity, the induction of immunogenic cell death (ICD) by second-generation photosensitizers, has shown limited efficacy in desmoplastic tumor models, including PDAC. This limitation arises from poor tissue penetration and the inability to sustain robust ROS levels required to trigger effective adaptive immune responses.^27–31^

In this context, the observation of calreticulin exposure following treatment with PS2 suggests that this compound offers clear advantages over conventional photosensitizers. Notably, PS2 was able to elicit ICD signals even within a notoriously refractory microenvironment. This characteristic places PS2 closer to the profile of ‘next-generation’ photosensitizers, in which the ability to stimulate antitumor immune responses is considered a highly desirable attribute—particularly for hard-to-treat tumors such as PDAC.

## 5 Conclusions

This study highlights the promising potential of the PS2 complex as an effective photosensitizer for photodynamic therapy against PDAC. Through a combination of favorable physicochemical properties—especially the presence of the ferrocene group and increased lipophilicity—this study highlights the promising potential of the PS2 demonstrated higher cellular uptake, sustained and dose-dependent generation of ROS, and potent disruption of mitochondria and lysosomes in both homogeneous and heterogeneous three-dimensional tumor models. These effects translated into significant induction of apoptosis and signs of immunogenic cell death, including calreticulin exposure and damage to essential organelles.

## Supplementary Material



## Notes

Experimental details; synthesis protocols; supplementary methods; fluorescence microscopy images; dose–response curves; ROS generation kinetics; and organelle staining assays (MitoTracker and LysoTracker). Supplementary figures provide representative microscopy images and quantitative analyses for homogeneous and heterogeneous 3D pancreatic spheroid models (PDF)
