# Supplementary material for: Palladium(II)-Complexed meso-Tetra(4-pyridyl)porphyrin: Photodynamic Efficacy in 3D Pancreatic Cancer Models
Source: ACS Omega. 2025 Oct 30;10(44):53564–71. doi: 10.1021/acsomega.5c09619 (PMC12613124; doi:10.1021/acsomega.5c09619)
Supplement: Supplementary file 1 [file ao5c09619_si_001.pdf]

SUPPORTING INFORMATION

Palladium(II)-Complexed meso-Tetra(4-pyridyl)porphyrin: Photodynamic Efficacy in 3D Pancreatic Cancer Models

Edynara Cruz de Moraes[1*], Lívia do Carmo Silva[2], Juliana Santana de Curcio[2], Alex Marchezini Graça[3], Alzir Azevedo Batista[3], Elisângela de Paula Silveira Lacerda[2], Pablo José Gonçalves[1,4**].

1 Institute of Physics, Federal University of Goiás, Goiânia, Goiás, 74690-900, Brazil.
2 Laboratory of Oncogenetics and Medical Genetics, Institute of Biological Sciences, Federal University of Goiás, Goiânia, Goiás, 74690-900, Brazil.
3 Department of Chemistry, Federal University of São Carlos (UFSCar), São Carlos, São Paulo, 13565-905, Brazil.
4 Center of Excellence in Hydrogen and Sustainable Energy Technologies (CEHTES), Goiânia, Goiás, 74690-900, Brazil.

Corresponding author: (*) E.C. Moraes (edynaramoraes@discente.ufg.br) (**) P.J. Gonçalves (pablo@ufg.br)

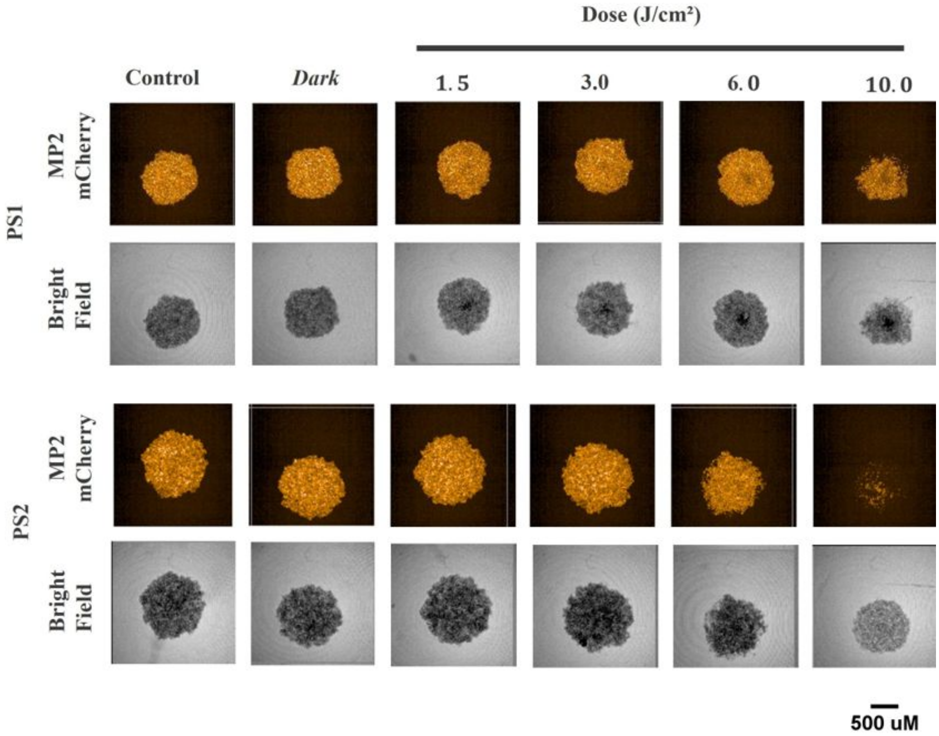

Figure S1: Fluorescence (mCherry) and bright field images of homogeneous MIA PaCa-2 spheroids treated with the compounds PS2 and PS1 at a concentration of 1 μM, followed by irradiation with different light doses (1.5, 3, 6, and 10 J/cm²). Control (untreated) and dark (treated without light) groups were included for comparison.

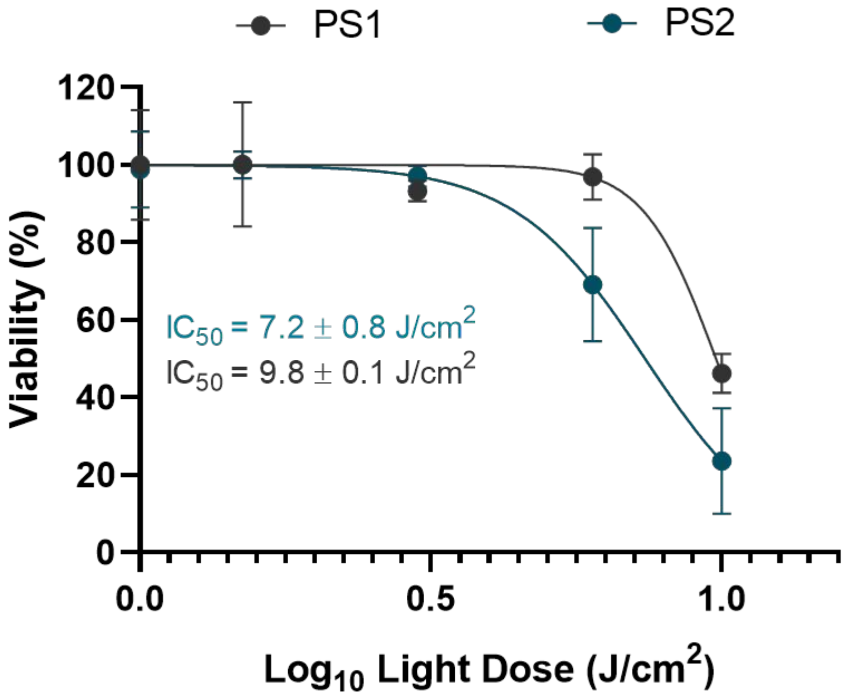

Figure S2: Dose-response curve of photodynamic therapy. Quantification of mCherry fluorescence intensity in homogeneous MIA PaCa-2 spheroids treated with PS1 and PS2 at a concentration of 1 µM, after irradiation with increasing light doses (1.5, 3, 6, and 10 J/cm²). Fluorescence values were normalized to the dark group

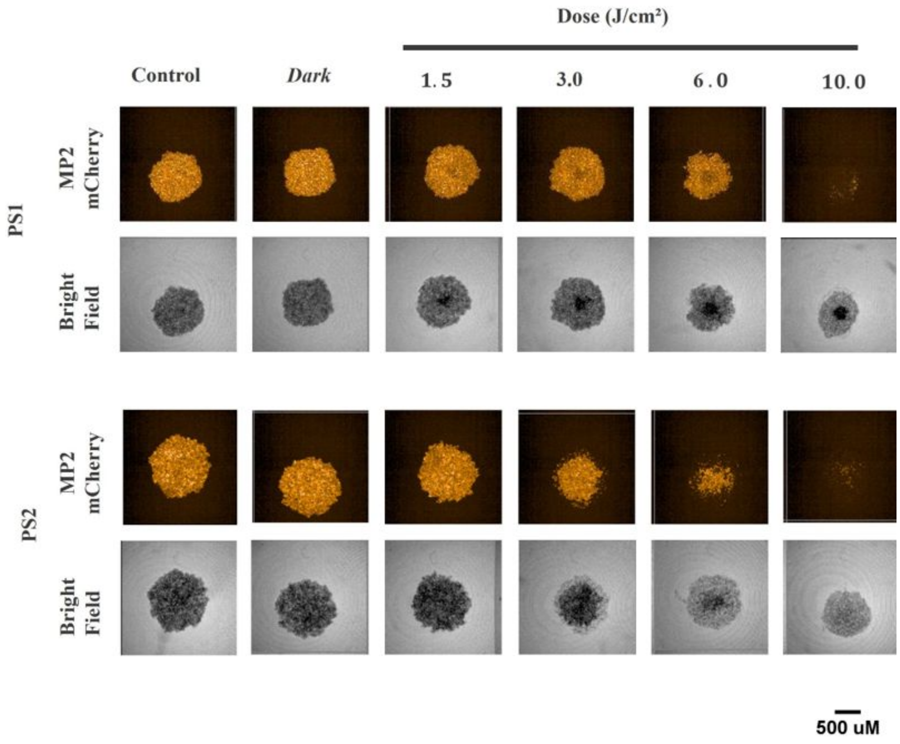

Figure S3: Fluorescence (mCherry) and bright field images of homogeneous MIA PaCa-2 spheroids treated with the compounds PS2 and PS1 at a concentration of 2 µM, followed by irradiation with different light doses (1.5, 3, 6, and 10 J/cm²). Control (untreated) and dark (treated without light) groups were included for comparison.

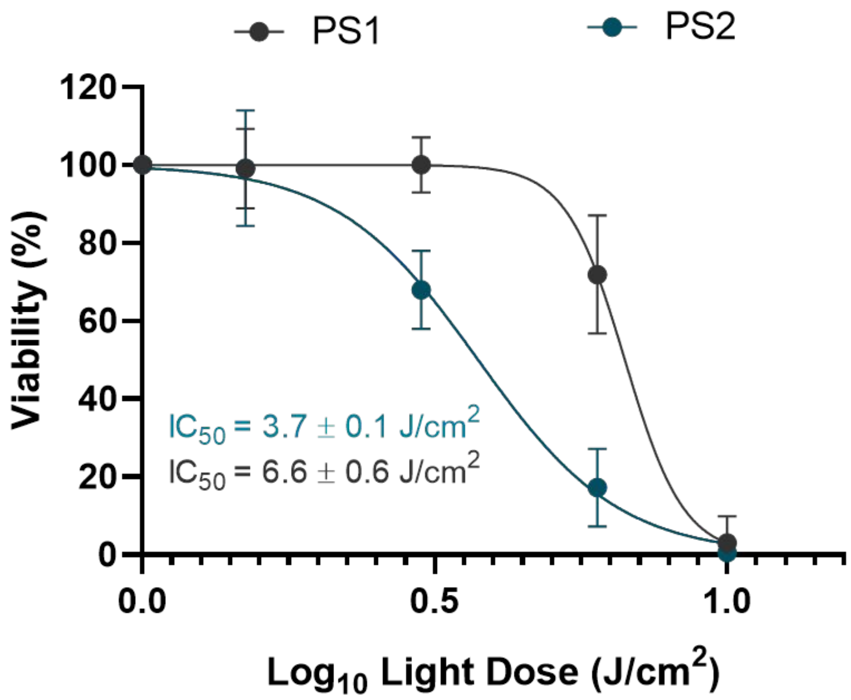

Figure S4: Dose-response curve of photodynamic therapy. Quantification of mCherry fluorescence intensity in homogeneous MIA PaCa-2 spheroids treated with PS1 and PS2 at a concentration of 2 µM, after irradiation with increasing light doses (1.5, 3, 6, and 10 J/cm²). Fluorescence values were normalized to the dark group

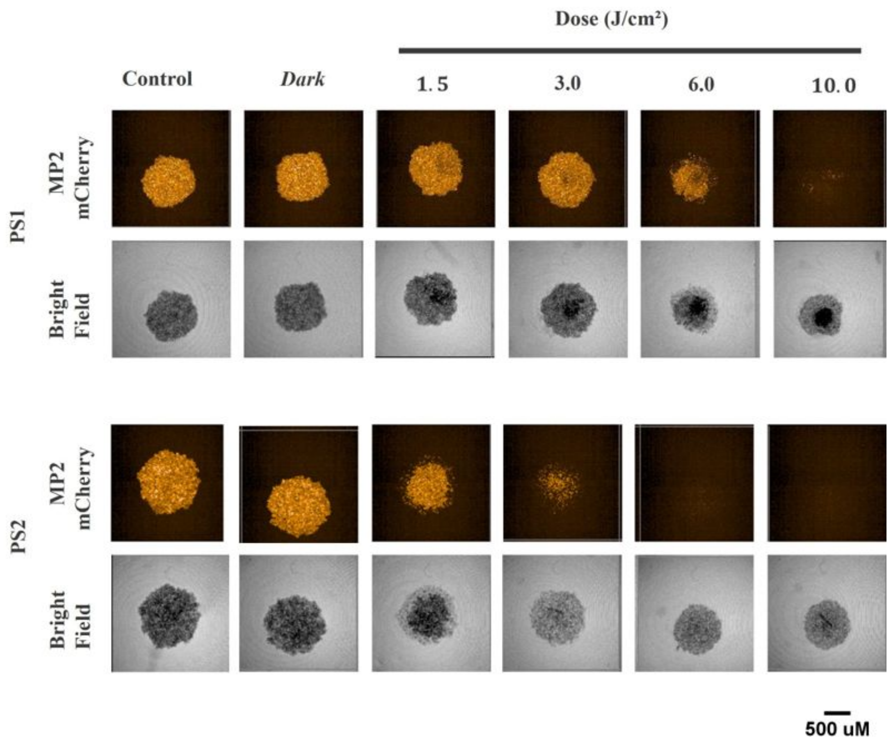

Figure S5: Fluorescence (mCherry) and bright field images of homogeneous MIA PaCa-2 spheroids treated with the compounds PS1 and PS2 at a concentration of 3 μM, followed by irradiation with different light doses (1.5, 3, 6, and 10 J/cm²). Control (untreated) and dark (treated without light) groups were included for comparison.

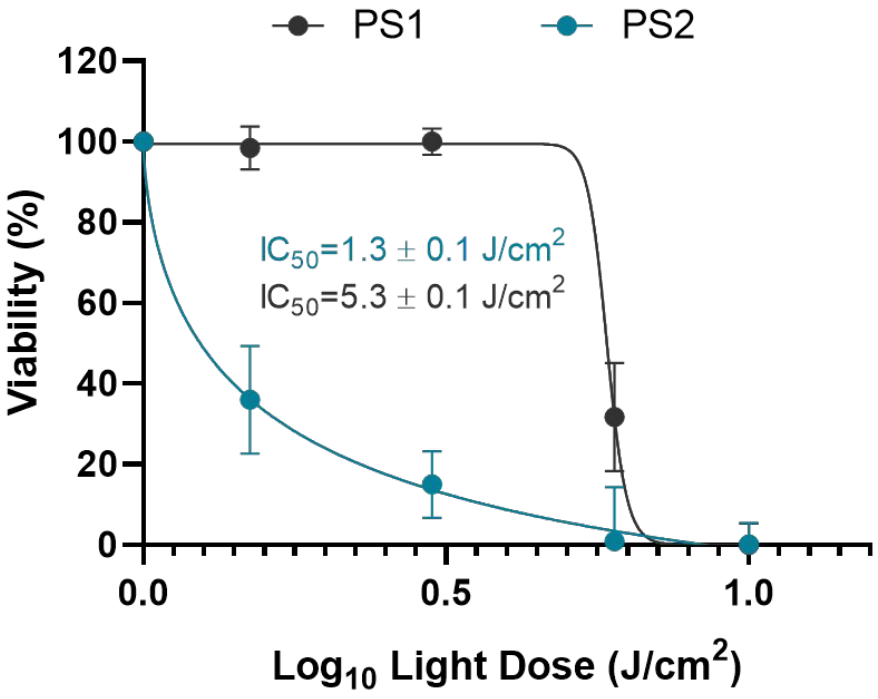

Figure S6: Dose-response curve of photodynamic therapy. Quantification of mCherry fluorescence intensity in homogeneous MIA PaCa-2 spheroids treated with PS2 and PS1 at a concentration of 3 μM, after irradiation 1.5, 3, 6, and 10 J/cm². Fluorescence values were normalized to the dark group. Doses (1.5, 3, 6, and 10 J/cm²) correspond to the fluence applied during PDT and were used to assess dose-dependent photodynamic effects. These values do not represent IC_{50} concentrations.

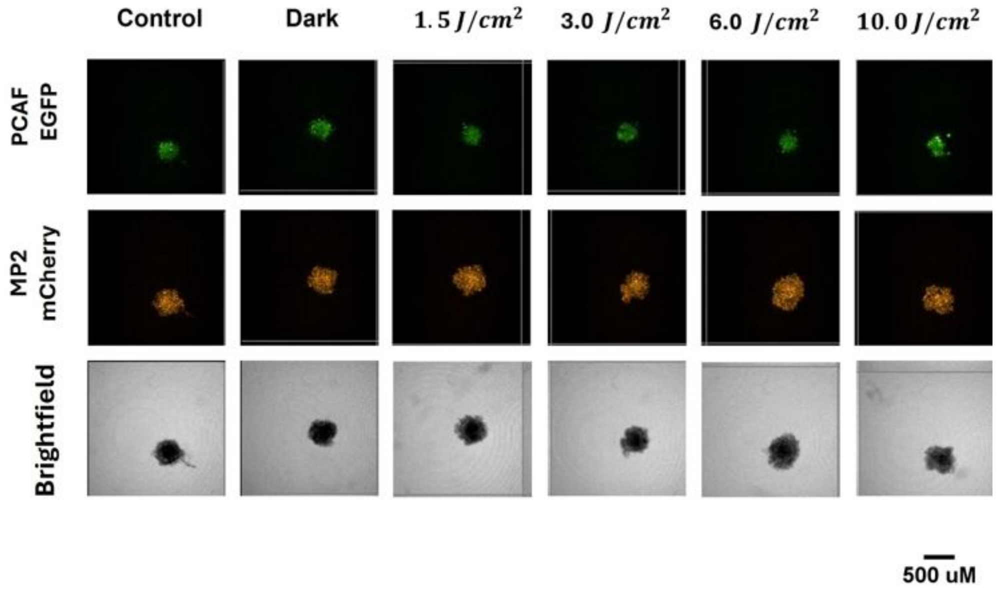

Figure S7: Fluorescence (mCherry) and bright field images of heterogeneous MIA PaCa-2 spheroids. Heterogeneous spheroids composed of mCherry-expressing MIA PaCa-2 cells co-cultured with EGFP-expressing PCAFs were treated with the PS2 at a concentration of 1 μM, followed by irradiation with different light doses (1.5, 3, 6, and 10 J/cm²). Control (untreated) and dark (treated without light) groups were included for comparison.

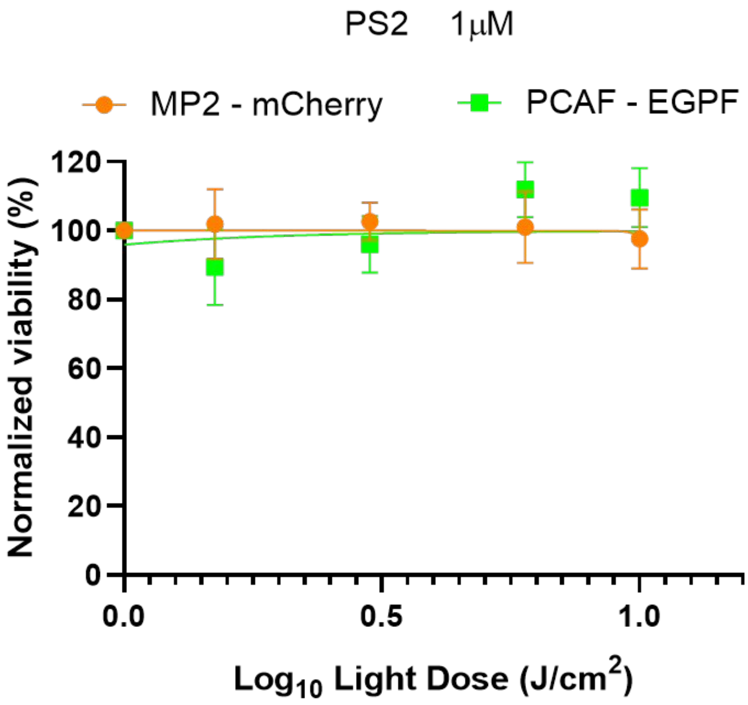

Figure S8: Dose-response curve of photodynamic therapy. Quantification of mCherry fluorescence intensity in heterogeneous MIA PaCa-2 spheroids co-cultured with PCAFs and treated with PS1 and PS2 at a concentration of 1 μM, after irradiation with increasing light doses (1.5, 3, 6, and 10 J/cm²). Fluorescence values were normalized to the dark group.

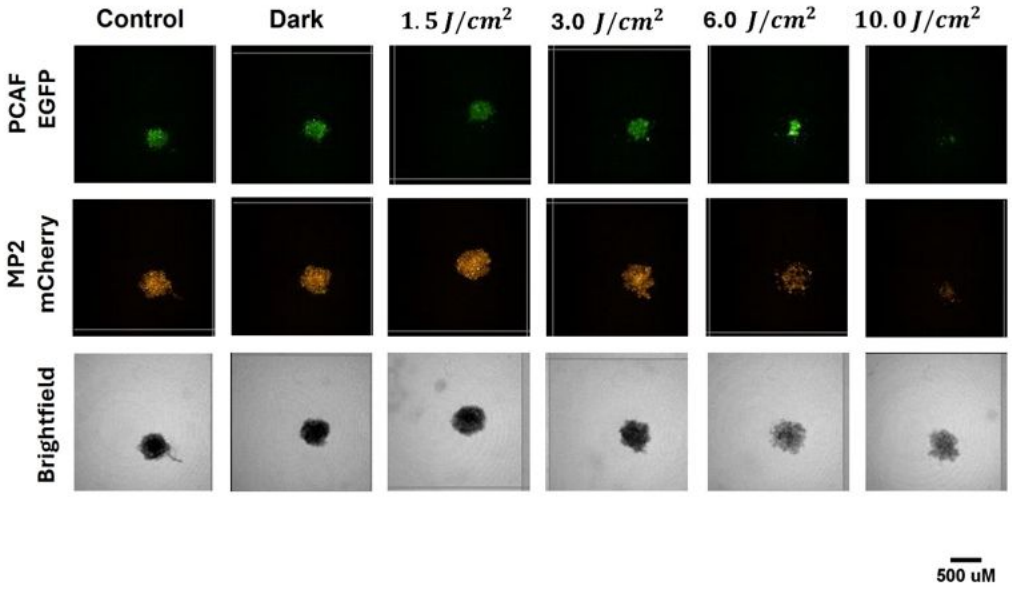

Figure S9: Fluorescence (mCherry) and bright field images of heterogeneous MIA PaCa-2 spheroids. Heterogeneous spheroids composed of mCherry-expressing MIA PaCa-2 cells co-cultured with EGFP-expressing PCAFs were treated with the PS2 at a concentration of 2 μM,

followed by irradiation with different light doses (1.5, 3, 6, and 10 J/cm²). Control (untreated) and dark (treated without light) groups were included for comparison.

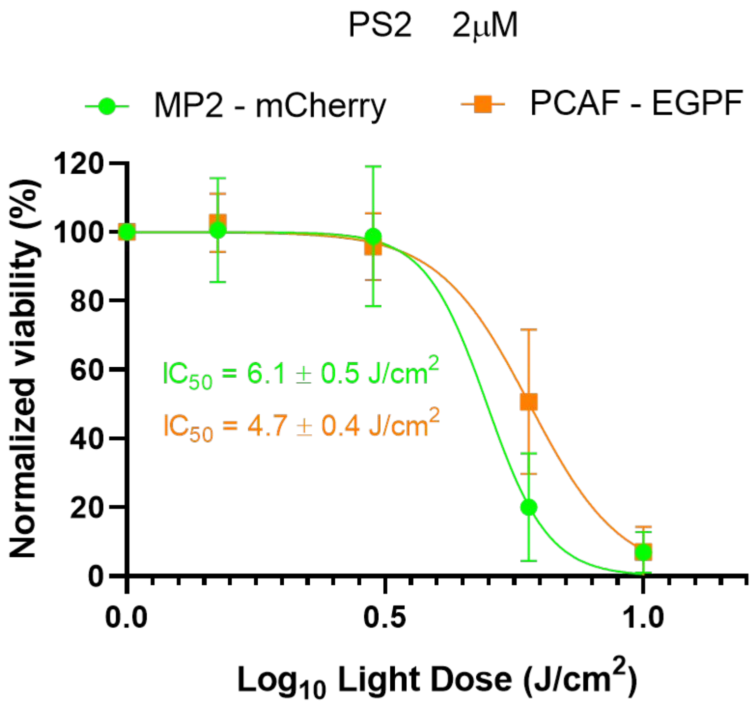

Figure S10: Dose-response curve of photodynamic therapy. Quantification of mCherry fluorescence intensity in heterogeneous MIA PaCa-2 spheroids co-cultured with PCAFs and treated with PS2 at a concentration of 2 μM, after irradiation with increasing light doses (1.5, 3, 6, and 10 J/cm²). Fluorescence values were normalized to the dark group.

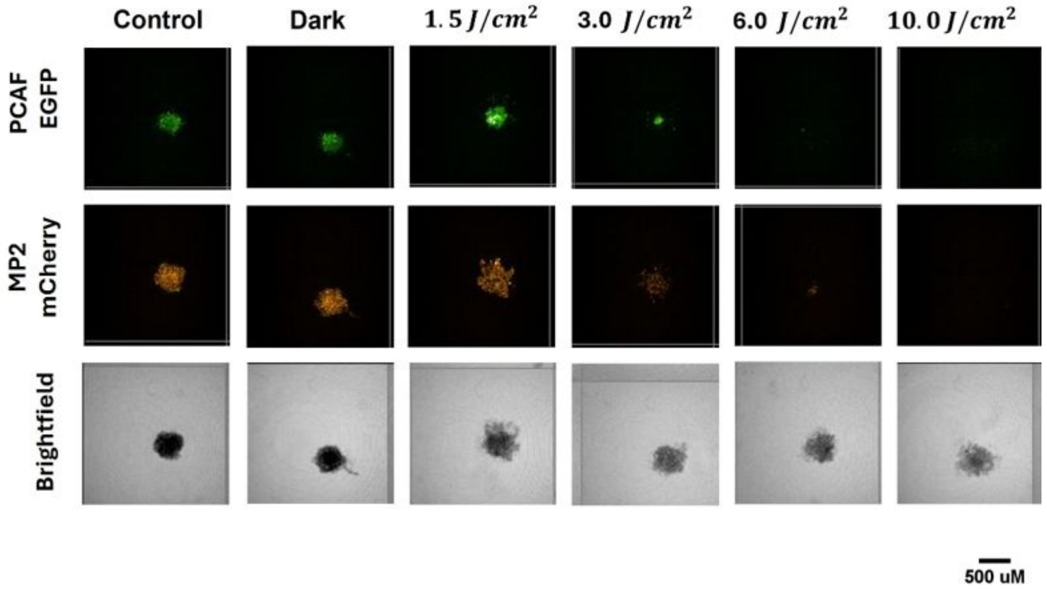

Figure S11: Fluorescence (mCherry) and bright field images of heterogeneous MIA PaCa-2 spheroids. Heterogeneous spheroids composed of mCherry-expressing MIA PaCa-2 cells co-cultured with EGFP-expressing PCAFs were treated with the PS2 at a concentration of 3 µM, followed by irradiation with different light doses (1.5, 3, 6, and 10 J/cm²). Control (untreated) and dark (treated without light) groups were included for comparison.

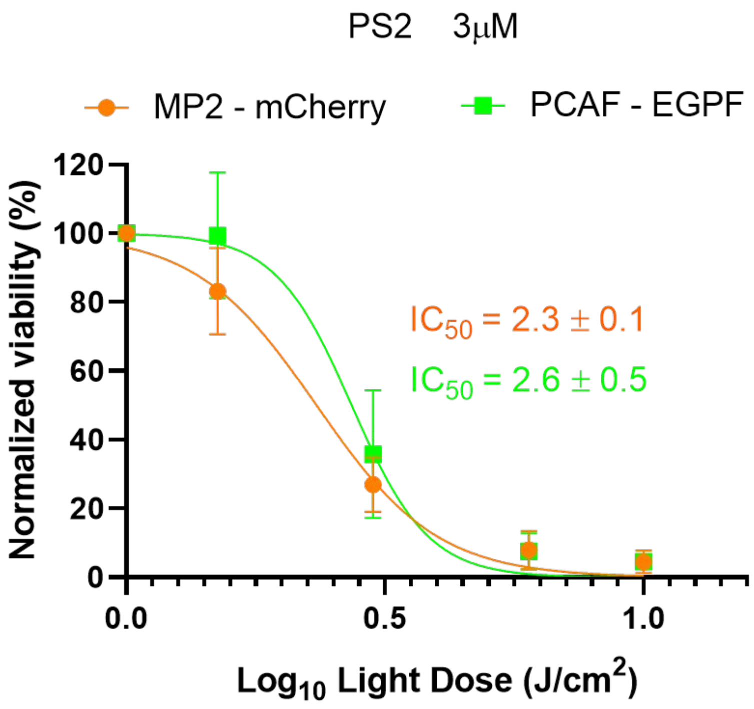

Figure 12: Dose-response curve of photodynamic therapy. Quantification of mCherry fluorescence intensity in heterogeneous MIA PaCa-2 spheroids co-cultured with PCAFs and treated with PS2 at a concentration of 3 µM, after irradiation with increasing light doses (1.5, 3, 6, and 10 J/cm²). Fluorescence values were normalized to the dark group.
